# A Novel Apoptosis Correlated Molecule: Expression and Characterization of Protein Latcripin-1 from *Lentinula edodes* C_91–3_

**DOI:** 10.3390/ijms13056246

**Published:** 2012-05-21

**Authors:** Ben Liu, Mintao Zhong, Yongzhi Lun, Xiaoli Wang, Wenchang Sun, Xingyun Li, Anhong Ning, Jing Cao, Wei Zhang, Lei Liu, Min Huang

**Affiliations:** Department of Microbiology, Dalian Medical University, No. 9 West Section, Lvshun South Road, Lvshunkou District, Dalian 116044, China; E-Mails: kinglb@sina.com (B.L.); dalianzhongmintao@163.com (M.Z.); lunyz@163.com (Y.L.); vacancy@sina.com (X.W.); wenchangsun@yahoo.com (W.S.); lxy0605@sina.com (X.L.); dlmedu@dl.cn (A.N.); caojingaizhe@163.com (J.C.); wisherzhw2007@163.com (W.Z.); liulei875@163.com (L.L.)

**Keywords:** *Lentinula edodes*, apoptosis, protein Latcripin-1, *Pichia pastoris*, expression

## Abstract

An apoptosis correlated molecule—protein Latcripin-1 of *Lentinula edodes* C_91–3_—was expressed and characterized in *Pichia pastoris* GS115. The total RNA was obtained from *Lentinula edodes* C_91–3_. According to the transcriptome, the full-length gene of Latcripin-1 was isolated with 3′-Full Rapid Amplification of cDNA Ends (RACE) and 5′-Full RACE methods. The full-length gene was inserted into the secretory expression vector pPIC9K. The protein Latcripin-1 was expressed in *Pichia pastoris* GS115 and analyzed by Sodium Dodecylsulfonate Polyacrylate Gel Electrophoresis (SDS-PAGE) and Western blot. The Western blot showed that the protein was expressed successfully. The biological function of protein Latcripin-1 on A549 cells was studied with flow cytometry and the 3-(4,5-Dimethylthiazol-2-yl)-2,5-Diphenyl-tetrazolium Bromide (MTT) method. The toxic effect of protein Latcripin-1 was detected with the MTT method by co-culturing the characterized protein with chick embryo fibroblasts. The MTT assay results showed that there was a great difference between protein Latcripin-1 groups and the control group (*p* < 0.05). There was no toxic effect of the characterized protein on chick embryo fibroblasts. The flow cytometry showed that there was a significant difference between the protein groups of interest and the control group according to apoptosis function (*p* < 0.05). At the same time, cell ultrastructure observed by transmission electron microscopy supported the results of flow cytometry. The work demonstrates that protein Latcripin-1 can induce apoptosis of human lung cancer cells A549 and brings new insights into and advantages to finding anti-tumor proteins.

## 1. Introduction

Throughout the world, cancer is one of the most serious diseases threatening the health of human beings [1–5]. It has become an urgent task to search for new drugs which have fewer side effects and which directly improve the survival rate of patients in the long term [6,7]. *Lentinula edodes* C_91–3_ is an edible mushroom isolated from Basidiomycetes Umbelliferae fungi. Since 1991, according to the international study of pharmaceutical research for *Lentinula Edodes*, six strains of mycels of *Lentinula edodes* have been fermented with the fermentation technology of bioengineering. One of the strain’s fermentation broths was found to have a direct anti-tumor effect [8,9]. Because the strain was studied beginning in March 1991, it was named as “C_91–3”_. “C” represents “China”. The extracts contain a variety of proteins in addition to polysaccharides and amino acids [10–12]. The *in vivo* and *in vitro* experiments confirmed that some of the protein components have significant effects on inducing cell apoptosis [10,13]. According to the transcriptome sequence, we designed primers and used the 3′-Full RACE and 5′-Full RACE methods to produce the full-length gene. We induced and expressed the protein with the *Pichia pastoris* expression system and incubated the human lung cancer cell A549 with the identified protein. The biological function of protein Latcripin-1 on A549 cells was studied with flow cytometry and the 3-(4,5-Dimethylthiazol-2-yl)-2,5-Diphenyl-tetrazolium Bromide (MTT) method. Finally, we analyzed whether the protein had the function of inducing apoptosis.

## 2. Results and Discussion

### 2.1. CDS Region of *Lentinula edodes* C_91–3_ Latcripin-1 Full-Length Gene

To test the anti-tumor protein in *Lentinula edodes* C_91–3_, the total RNA of *Lentinula edodes* C_91–3_ was extracted and a cDNA library was prepared. The short reads were obtained by using the Solexa High-flux Sequencing Technique. These short reads were assembled *de novo* with the sequence assembly software SOAP2. The output was the Unigene sequences, which could not be extended at both ends. At the same time, the given information was parsed and the gene function was annotated [14]. The Unigene sequences were blasted with the blastx method. All of the information on the *Lentinula edodes* C_91–3_ transcriptome was obtained. Latcripin-1 was found to have an apoptosis correlated function in the Cluster of Orthologous Groups of proteins (COG) database (COG-ID: COG5032). According to the preliminary results of the gene functional annotation, the domains were analyzed using the online tools Sanger Pfam. Finally, the apoptosis correlated molecule-Latcripin-1 was screened out of Unigene sequences. Latcripin-1 was named from three words: *Lentinula edodes*, transcriptome and protein. The stem “La tcri pin” represented the novel molecule.

**Figure 1:**
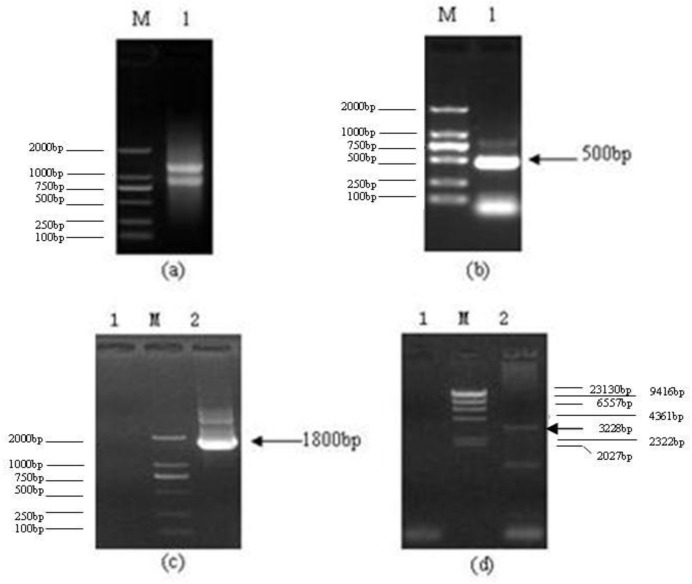
The results of agarose gel electrophoresis. (**a**) Total RNA of *Lentinula edodes* C_91–3_. M: DNA Marker DL2000. 1: total RNA of *Lentinula edodes* C_91–3_. (**b**) 3′-Full RACE reaction of Latcripin-1 gene. M: DNA Marker DL2000. 1: product of 3′-Full RACE inner PCR. (**c**) 5′-Full RACE reaction of Latcripin-1 gene. M: DNA Marker DL2000. 1: product of 5′-Full RACE inner PCR (M-MLV(−)). 2: product of 5′-Full RACE inner PCR (M-MLV (+)). (**d**) Full-length of Latcripin-1 gene. M: DNA Marker λ-Hind III digest. 1: product of RT-PCR (M-MLV (−)). 2: product of RT-PCR (M-MLV (+)).

**Figure 2:**
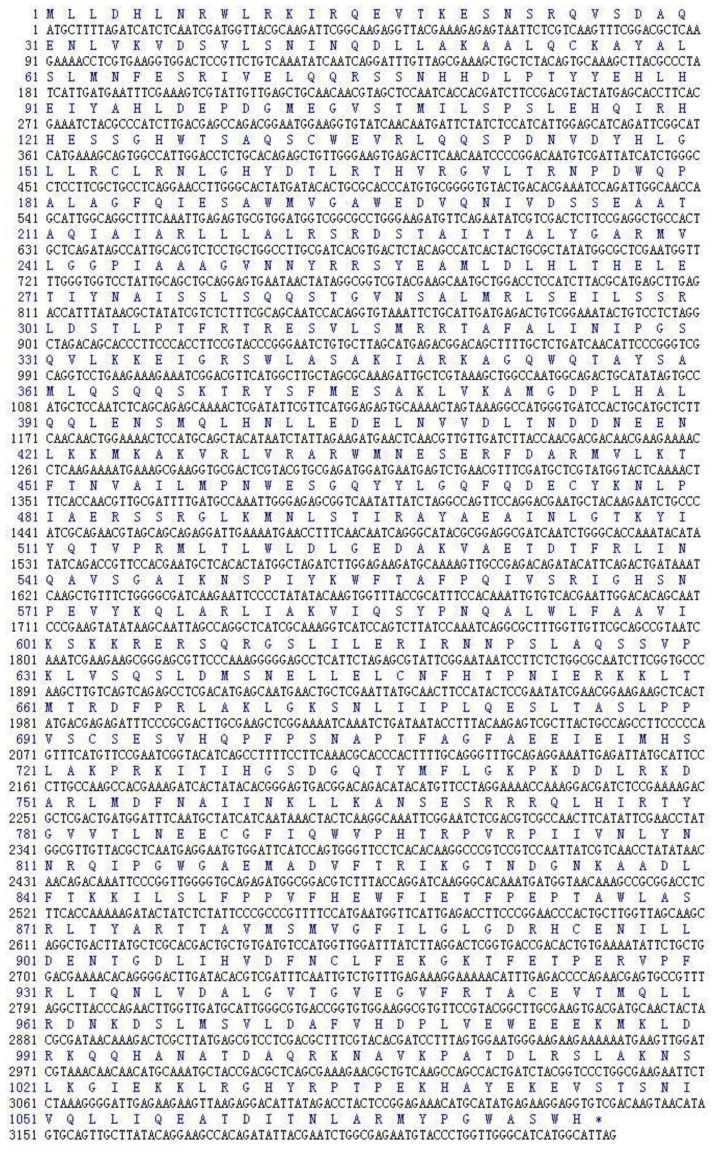
The full-length CDS region of *Lentinula edodes* C_91–3_ Latcripin-1 gene and the amino acid sequence.

Three microliters of Total RNA of *Lentinula edodes* C_91–3_ and 5 μL of marker were added into 1% agarose gel. It was indicated that the total RNA was complete (Figure 1a). After the 3′-Full RACE reaction, 5 μL Inner Polymerase Chain Reaction (PCR) product and 5 μL marker were added into 1.5% agarose gel. A clear band was visible at 500 bp (Figure 1b). After the 5′-Full RACE reaction, 5 μL Inner PCR product and 5 μL marker were added to 1.5% agarose gel. A clear band appeared at 1800 bp, while no bands appeared in the M-MLV (−) control group (Figure 1c). After the RT-PCR reaction with primer F and primer R, 5 μL RT-PCR product and 5 μL marker were added to 1.0% agarose gel for electrophoresis. Above 3000 bp a clear band was shown. The band size was consistent with the sequencing results, while there were no bands in the M-MLV (−) control group (Figure 1d). The *Lentinula edodes* C_91–3_ Latcripin-1 full-length gene Coding Sequence (CDS) region is as follows (Figure 2) (GenBank Accession Number: JQ327159). The amino acid sequence of protein Latcripin-1 was obtained according to the gene sequence (Figure 2).

### 2.2. Amino Acid Sequence Analysis of Protein Latcripin-1

**Figure 3:**
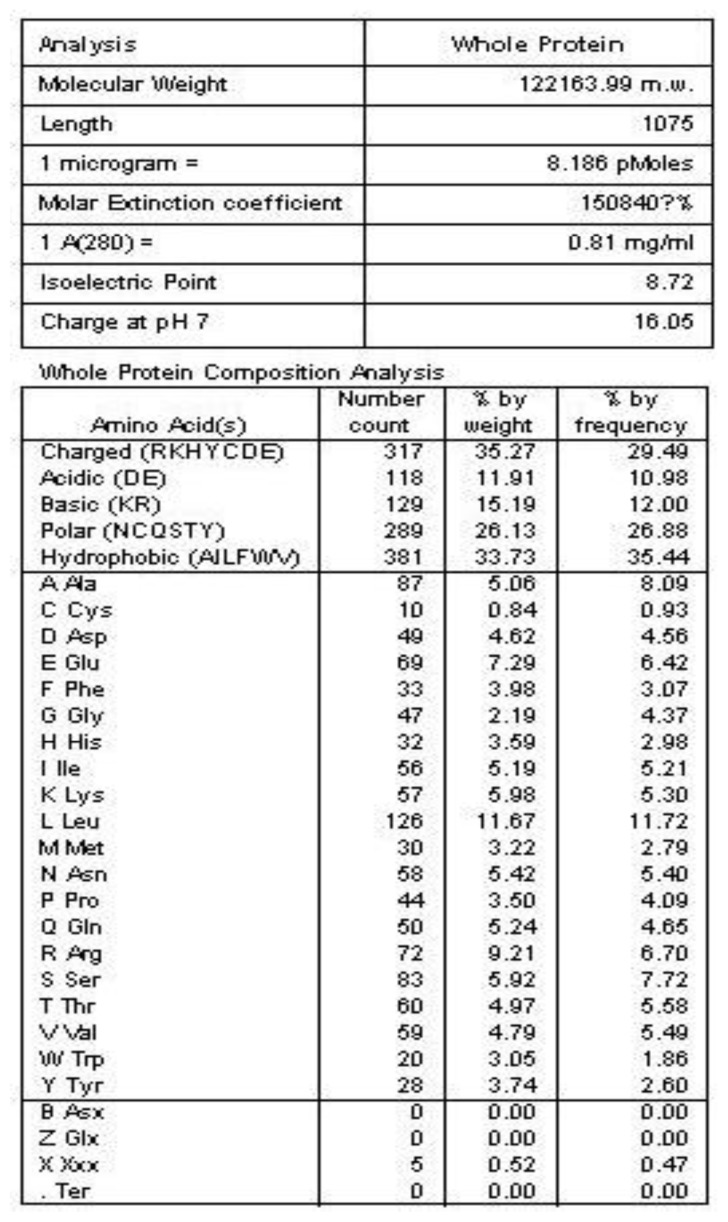
The general amino acid analysis of protein Latcripin-1.

**Figure 4:**
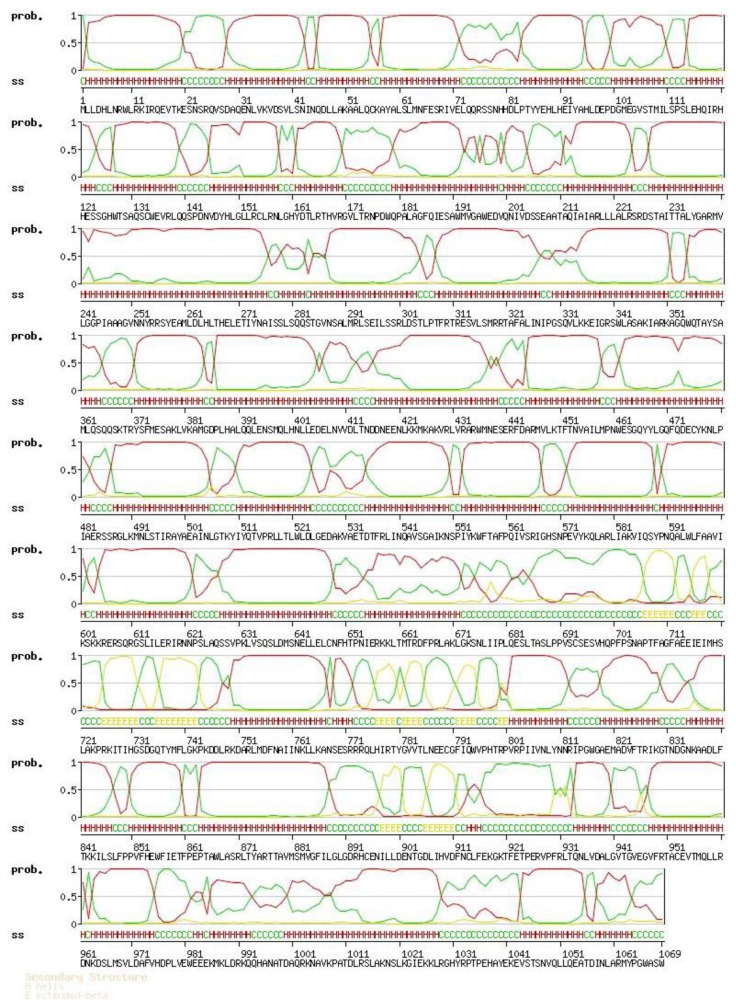
The secondary structure of protein Latcripin-1. H: helix; E: extended-beta; C: coil.

**Figure 5:**
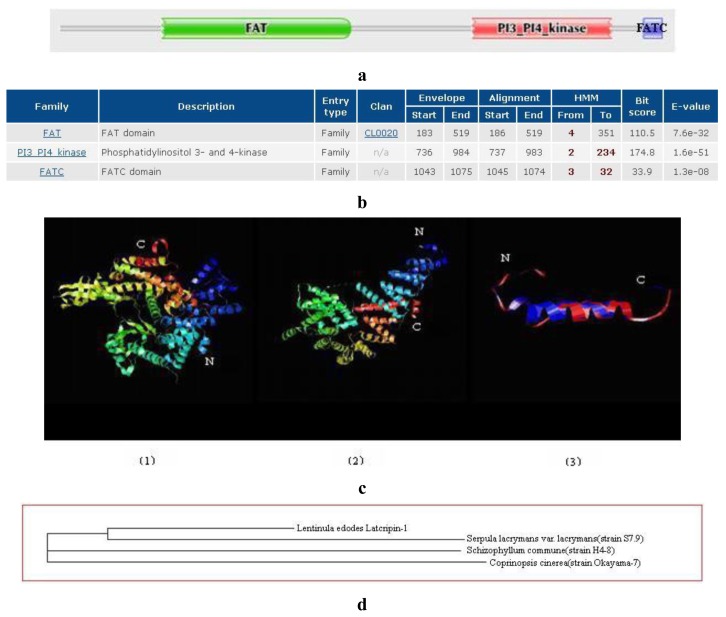
(**a**) The structural domain analysis of protein Latcripin-1 with Pfam database (Significant Pfam-A Matches). (**b**) The location of the amino acids structural domain. (**c**) The tertiary structure of protein Latcripin-1. (1) FAT domain; (2) PI3K kinase domain; (3) FATC domain. (**d**) The cladogram depicting of protein Latcripin-1.

The general amino acid analysis of protein Latcripin-1 was done by DNAStar software [15] (Figure 3). The secondary structure of protein Latcripin-1 was analyzed with the Swiss-Model database [16] (Figure 4) and the Pfam database [17]. The results showed that three Pfam-A matched to the search sequence (all significant) (Figure 5a). The location of the amino acids spanning the different domains is shown in Figure 5b. After that, the tertiary structure of protein Latcripin-1 was predicted using homology structure modeling on the Swiss-Model server [16]. The tertiary structure of protein Latcripin-1 is between the Target of Rapamycin (TOR) and Phosphatidylinositol 3-kinase (PI3K) superfamily. There is a kinase domain in C-terminal, which is similar to the catalytic domain of PI3K (Figure 5c2). A FAT (for FRAP, ATM, TRAP) domain exists in other PIKK proteins families upstream of the kinase domain (Figure 5c1). At the end of the *C*-terminal, there is another FAT structure territory, called FATC (for FRAP, ATM, TRAP in *C*-terminal end) (Figure 5c3). Models of the functional domain of protein Latcripin-1 were colored from blue (*N* terminus) to red (*C* terminus). The modeling parameters are shown in Table 1. At the same time, the cladogram depicting of protein Latcripin-1 was analyzed with the multiple sequence alignment program ClustalW2. The similar proteins from *Serpula*, *Schizophyllum* and *Coprinopsis* species (Identity > 50%) were aligned (Figure 5d). Protein Latcripin-1 is more homologous to *Serpula* species than to the other two.

### 2.3. Transformation of *Pichia pastoris* GS115 and Selection of Transformants

**Figure 6:**
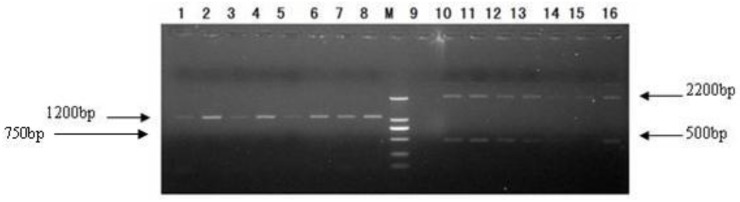
Selection of transformants. **1**–**8**: GS115–pPIC9K/Latcripin-1 clone (R1seq primer and 5′AOX I primer). M: DL2000 DNA Marker. **9**: GS115–pPIC9K clone (R1seq primer and 5′AOX I primer). **10**–**16**: GS115- pPIC9K clone (3′AOX I primer and 5′AOX I primer).

After PCR amplification, 5 μL PCR reaction solution was taken for 1.0% agarose gel electrophoresis. A 1200 bp band was seen in the GS115–pPIC9K/Latcripin-1 group. In the GS115–pPIC9K group 500 bp and 2200 bp bands were seen. There was no band in the GS115–pPIC9K group when gene-specific primers were used (Figure 6). The second characterized GS115–pPIC9K/Latcripin-1 clone was selected at random for further work.

### 2.4. Expression and Characterization of Protein Latcripin-1 in *Pichia pastoris* GS115

**Figure 7:**
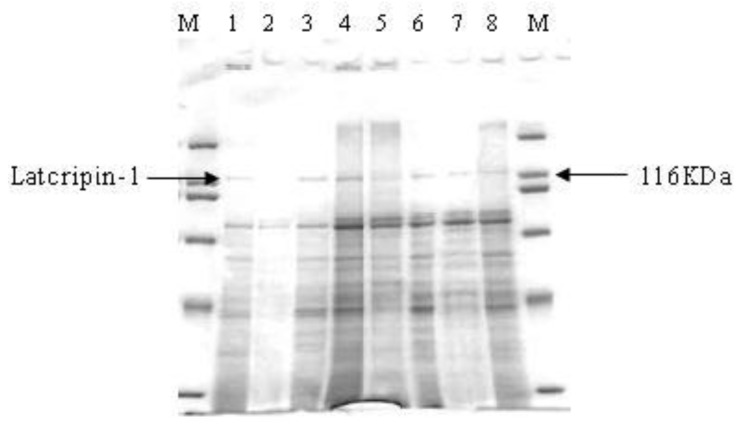
Sodium Dodecylsulfonate Polyacrylate Gel Electrophoresis (SDS-PAGE) of supernatants in different time spans. M: Protein Molecular Weight Marker (Broad). **1**: supernatants of GS115–Latcripin-1 (24 h). **2**: supernatants of GS115–pPIC9K (96 h). **3,4**: supernatants of GS115–Latcripin-1 (96 h). **5,6**: supernatants of GS115-Latcripin-1 (72 h). **7,8**: supernatants of GS115–Latcripin-1 (48 h).

Supernatants on different time spans (24 h, 48 h, 72 h, 96 h) were collected and analyzed by Sodium Dodecylsulfonate Polyacrylate Gel Electrophoresis (SDS-PAGE) (Figure 7). The sample volume was 25 μL and the marker volume was 5 μL. GS115–pPIC9K was used as a negative control. It was shown that the target protein appeared from 24 h until 96 h at 118 kda, while no interesting protein appeared in the negative control supernatant (96 h).

**Figure 8:**
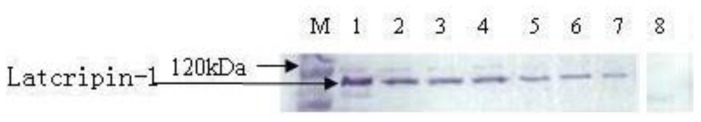
Western blot of protein Latcripin-1 in different time spans. M: Precision Plus Protein Standards Dual color (with 6× His tag). **1,2**: supernatants of GS115–Latcripin-1 (96 h). **3,4**: supernatants of GS115–Latcripin-1 (72 h). **5,6**: supernatants of GS115–Latcripin-1 (48 h). **7**: supernatants of GS115–Latcripin-1 (24 h). **8**: supernatants of GS115–pPIC9K (96 h).

To characterize the expressed protein, the Western blot method was used, the results of which are demonstrated in Figure 8. Anti-His-Tag positive bands appeared in the position of 118 kda. No band appeared in the negative control group.

### 2.5. Affinity Purification of Protein Latcripin-1 from *Pichia pastoris* Culture Supernatant

**Figure 9:**
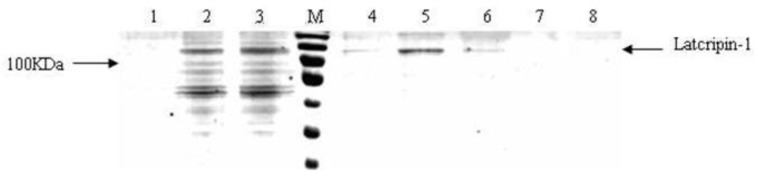
Affinity purification of protein Latcripin-1. M: PageRuler Prestained Protein Ladder. **1**: blank control. **2,3**: culture supernatants induced for 96 h. **4**: the first 1 mL eluted fraction. **5**: the second 1 mL eluted fraction. **6**: the third 1 mL eluted fraction. **7**: the fourth 1 mL eluted fraction. **8**: the fifth 1 mL eluted fraction.

The supernatant that cultured for 96 h was freeze-dried. The freeze-dried supernatant was dissolved into 20 mL pure water to be purified. Five milliliters resuspended protein was added to each column, respectively. The fractions of 5 mL Soluble Elution Buffer were collected into 5 Eppendorf tubes (1.5 mL) and analyzed by SDS-PAGE (Figure 9). Finally, the purified protein was diluted into 7.5 μg/mL, 15 μg/mL and 30 μg/mL for biological functional identification.

### 2.6. Effects of Protein Latcripin-1 on the Proliferation and Apoptosis of Lung Cancer A549 Cells

#### 2.6.1. MTT assay of Protein Latcripin-1 on Human Lung Cancer Cell A549

**Figure 10:**
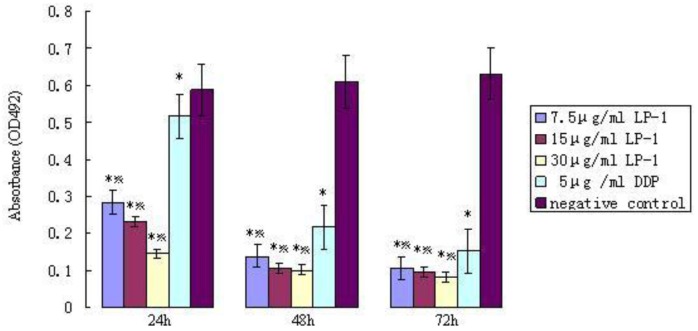
OD492 nm value of protein Latcripin-1 (LP-1) in different time spans of the MTT assay. * negative control group, *p* < 0.05. Five μg/mL DDP group, *p* < 0.05.

After 24 h of incubation, the cell inhibition of the purified protein group was higher than that of the control group. The difference of the OD492 nm value was significant (Figure 10). The growth of cancer cells A549 was inhibited significantly by protein Latcripin-1. In addition, the inhibition level of protein Latcripin-1 under different concentrations was higher than that of 5 μg/mL DDP (Cisplatin). We can see that the inhibiting function of protein Latcripin-1 on A549 cells was time and concentration dependent.

#### 2.6.2. Apoptosis Function Detection of Protein Latcripin-1 on Human Lung Cancer Cells A549

**Figure 11:**
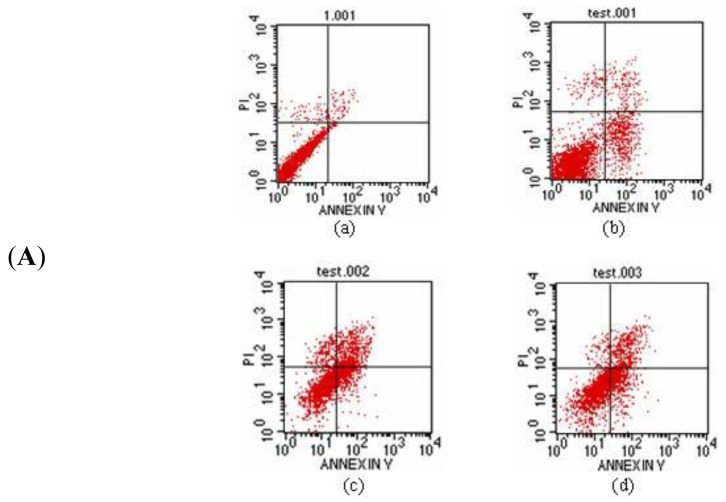

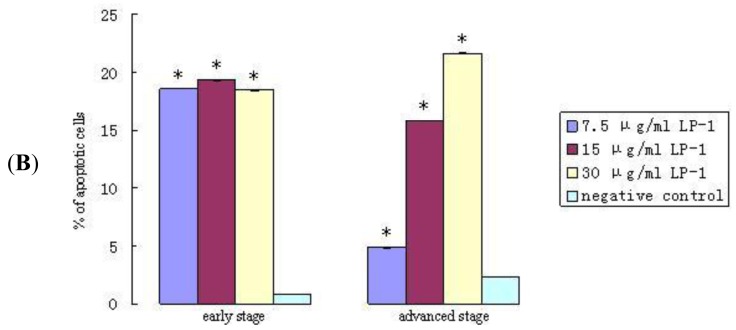
(**A**) The apoptosis rate of protein Latcripin-1 on A549 cell, (**a**) negative control group, (**b**) 7.5 μg/mL protein Latcripin-1 group, (**c**) 15 μg/mL protein Latcripin-1 group, (**d**) 30 μg/mL protein Latcripin-1 group; (**B**) The apoptosis rate of the expressed protein Latcripin-1 (LP-1) on A549 cell (%). * negative control, *p <* 0.05.

After 24 h of incubation, the A549 cells were stained with the Annexin/PI double staining method. The cell apoptosis rate was measured by flow cytometry in triplicate with the Cell Quest software [18] analysis and detection system (Figure 11a, b). In Figure 11a, the upper right quadrant shows the advanced stage apoptotic cells. The lower right quadrant shows early stage apoptotic cells. The upper left quadrant shows dead cells. The lower left quadrant shows normal cells. The results illustrate that when the concentration was increased to 30 μg/mL, the inducing apoptosis function of protein Latcripin-1 increased simultaneously. Under the interaction of protein Latcripin-1, the tolerance to physical and chemical effects of A549 cell was decreased, the cell debris was increased and the dead cells were increased significantly.

#### 2.6.3. Changes of Cell Ultrastructure Observed by Transmission Electron Microscopy (TEM)

**Figure 12:**
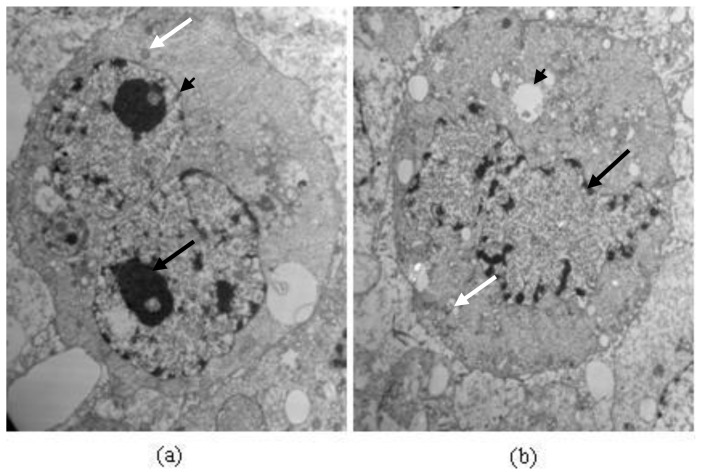
Electron micrographs of A549 cells. Magnification 8000×; TEM. (**a**) Cells of control group; and (**b**) cells treated with Protein Latcripin-1 (30 μg/mL) for 24 h.

TEM analysis exhibited different morphological alterations in A549 cells after treatment with protein Latcripin-1 (30 μg/mL) for 24 h (Figure 12). In the control group (Figure 12a), A549 cells maintained regular morphology with a large nucleus (black arrow), many mitochondria (white arrow), and membrane phase structural integrity (arrowhead). However, increase of intracytoplasmic vacuoles (arrowhead), chromatin condensation, nuclear fragmentation (black arrow), and mitochondrial swelling (white arrow) were observed in treated A549 cells (Figure 12b).

### 2.7. The Toxicity Test of Protein Latcripin-1 on Normal Chick Embryo Fibroblasts

**Figure 13:**
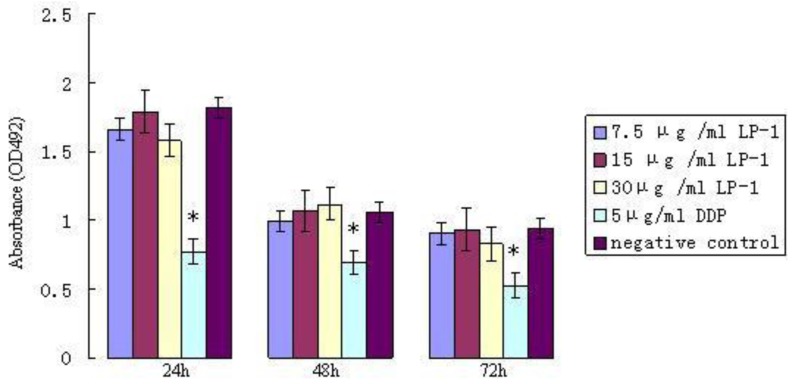
The toxic effect of protein Latcripin-1 (LP-1) on normal chick embryo fibroblasts in different time spans. * protein Latcripin-1 and negative control groups, *p <* 0.05.

The toxicity test results of protein Latcripin-1 on normal chick embryo fibroblasts (OD492 value, *χ̄* ± s) are shown in Figure 13. The growth of chick embryo fibroblasts was not inhibited with different concentrations of protein Latcripin-1. The difference was not significant compared with the negative control group. The cells were inhibited with 5 μg/mL DDP in the first 24 h of incubation. The difference was significant compared with the protein Latcripin-1 and negative control groups (*p <* 0.05).

### 2.8. Discussion

In this experiment, pPIC9K was chosen as the carrier. There is a strong promoter (AOXI) in it. With the promoter, pPIC9K can express the extraneous protein effectively under the induction of methanol [19–21]. pPIC9K is a shuttle plasmid. It cannot only replicate in prokaryotic cells, but can also be expressed in eukaryotic cells, which is convenient for experiments. The recipient strain used in this experiment is GS115, established by Cregg [22], which has the histidine dehydrogenase-deficient gene His4 (encoding histidine dehydrogenase gene). It can accept a vector with the His4 gene and turn into His+ phenotype transformants, and therefore can be screened in the histidine-deficient MD culture medium.

This is our first attempt to express the protein of *Lentinula edodes* C_91–3_ with the yeast expression system. *Pichia pastoris* has been well developed in recent years [23]. It is a methanol nutrition yeast expression system and is widely used for expressing extraneous proteins. As a type of eukaryon, *Pichia pastoris* not only can induce correct translation and post-processing of the extraneous eukaryon genes, but also has the function of modifying *N*- or *O*-glycosylation of the secreted protein. Recently, more and more exogenous genes were expressed successfully with this expression system: tumor necrosis factor [24], epidermal growth factor [25], human p53 protein [26], human serum albumin [27–29], human vascular endothelial cell growth factor [30] and human pro-insulin [31,32]. Results show that protein Latcripin-1 expressed through this system has high biological activity, which proves that the yeast expression system is suitable for the expression of fungus proteins.

This is also the first time we have separated the apoptosis-related proteins from *Lentinula edodes* C_91–3_. Apoptosis is an initiated death process during the cell ontogenesis process. The process is regulated by a series of proteins [33]. Through analyzing the protein domain of the Latcripin-1 gene, we found that the protein’s tertiary structure is very special. The structure is between TOR (target of rapamycin) and PI3K (phosphatidylinositol-3-kinase). There is a kinase domain in the *C*-terminal similar to the catalytic domain of PI3K. A FAT domain exists in other PIKK proteins families upstream of the kinase domain. PI3Ks are a family of enzymes involved in cellular functions such as cell growth, proliferation, differentiation, motility, survival and intracellular trafficking, which in turn are involved in cancer. In response to lipopolysaccharide, PI3K phosphorylates p65 induce anandamide synthesis to inhibit NF-κB activation. This is under the control of fatty acid amide hydrolase (FAAH). FAAH can limit the ability of lipase to increase AEA. This is also inhibited by wortmannin and cannabidiol, which are two of the only natural compounds to inhibit FAAH [34–36]. Many PIK-related proteins are involved in cell-cycle checkpoint controls (e.g., ATM, ATR, DNA-PK, ESR1 and Rad 3). Dysfunction can result in a range of diseases, including immunodeficiency, neurological disorder and cancer [37]. Also, it has been reported that the main pathways related to the pathogenesis of lung cancer are the PI3K/Akt-mTOR pathway and the Ras-Raf-MEK/ERK pathway [38]. At the end of the *C*-terminal, there is a FATC structure, which is considered to interact with the FAT to expose the kinase domain [39,40]. The FAT domain is named after FRAP, ATM and TRRAP. It mediates signaling through Grb2 via phosphorylated Y925. Bosotti [37] reported two crystal structures of the FAT domain. Large rearrangements of the structure are indicated to allow phosphorylation of Y925 and subsequent interaction with Grb2. The FATC domain is named after FRAP, ATM, TRRAP *C*-terminal [37]. The FAT domain is only present in the FRAP, ATM and TRRAP subfamilies and always coexists with the FATC domain [37]. The solution structure of the FATC domain suggests it plays a role in redox-dependent structural and cellular stability [41].

Thus, we speculated that such a molecular structure augments its biological activity in some aspect. It can also be seen from Figures 10, 11, 12 that the biological function of protein Latcripin-1 is significant. A549 cells were markedly induced to apoptosis. It was interesting to note that there was no toxic effect of the characterized protein on chick embryo fibroblasts. We hypothesized that there was no recognition site of protein Latcripin-1 in the chick embryo fibroblasts. However, further research is required to prove these hypotheses. At the same time, further research is required to elucidate the relationship of these functional domains. The improved understanding of these functional domains will potentially provide further understanding of the apoptosis mechanism of protein Latcripin-1.

## 3. Experimental Section

### 3.1. Strains, Plasmids, and Reagents

The *Escherichia coli* strain DH5a, used as the host for cloning the gene Latcripin-1, was equipped in our laboratory. The pPIC9K vector and the *Pichia pastoris* strain GS115 (his-mut+), used for protein Latcripin-1 expression, were from Invitrogen Co. (Beijing, China). The In-Fusion™ Advantage PCR Cloning Kit, 3′-Full RACE Core Set Ver2.0, 5′-Full RACE Kit, BigDye Terminater V3.1 Cycle Sequencing Kit, Plasmid purification kit, Lysis Buffer for Microorganism to Direct PCR, restriction polymerases and Primers were from Takara (Dalian, China). Trizol, Penta. His Antibody and HRP-Rabbit Anti-Mouse IgG (H + L) were from Invitrogen Co. (Beijing, China). Bicinchoninic Acid Kit for Protein Determination was from Sigma-Aldrich Trading Co., Ltd (Shanghai, China). Annexin V-FITC/PI kit was from Becton Dickinson Medical Devices Co., Ltd (Shanghai, China). The integrated potato culture medium was prepared by our own laboratory.

### 3.2. Method of Getting the CDS Region of *Lentinula edodes* C_91–3_ Latcripin-1 Full-Length Gene

The mycelium of *Lentinula edodes* C_91–3_ was cultured with integrated potato culture medium for 18 days in a mortar. It was then tritumed with liquid nitrogen. Total RNA was extracted with Trizol reagent. The obtained RNA was dissolved in 50 μL DEPC treated water. Primers of 3′-RACE and 5′-RACE experiments were designed with oligo6.0 software according to the transcriptome sequence results, all primers required for the experiments are listed as follows (Table 2).

The results of 3′-Full RACE and 5′-Full RACE were sequenced and stitched. Afterwards, primer F and primer R were designed accordingly. The cDNA was synthesized with Tkara 3′-Full Race Core Ver.2.0. The M-MLV (−) control was established at the same time. SnaB I/Not I restriction sites were added to the *N*- and *C*-terminal, respectively, in order to implement In-Fusion™ ligation [42]. A 6× HIS tag was added at the 3′end for later characterization. The cDNA was amplified by PCR with primer F and primer R. The sequence of the amplified gene was analyzed and confirmed by ABI PRISMTM 3730XL DNA Sequencer and Applied Biosystem.

### 3.3. Construction of Expression Vector Latcripin-1

The yeast eukaryotic expression vector, plasmid pPIC9K, was cut by SnaB I/Not I in 100 μL system at 37 °C for 4 h. The products were purified with Takara DNA Fragment Purification Kit Ver. 2.0, and then recycled with Takara Agarose Gel DNA Purification Kit Ver. 2.0. In order to identify, separate and purify the protein, a 6× His-tag was added at the end of Latcripin-1 gene. We used an In-Fusion™ Advantage PCR Cloning Kit for In-Fusion cloning in order to ligate the Latcripin-1 gene and expression vector. The constructed plasmid Latcripin-1 was transformed into a competent *E. coli* DH5a strain, which was spread on LB agar plates (pH 7.0) with 100 g/mL ampicillin and incubated overnight. The expression plasmid Latcripin-1 was obtained from the screened positive transformants and verified by both BamHI and NotI digestion. Sequencing was conducted at Takara Dalian Co.

### 3.4. Transformation of *Pichia pastoris* GS115 and Selection of Transformants

*Pichia pastoris* GS115 was transformed with the linearized expression vector Latcripin-1 by digestion with SacI. After lectrotransformation, the transformant cells were plated on RDB plates, incubated at 30 °C for 72 h. Meanwhile, a simple vector pPIC9K was transformed into *Pichia pastoris* GS115 as a negative control. The two different pastoris were named as GS115–pPIC9K/Latcripin-1 and GS115–pPIC9K, respectively. To select positive clones for protein expression, eight of the GS115–Latcripin-1 colonies and seven GS115–pPIC9K clones were chosen and amplificated (Lysis Buffer for Microorganism to Direct PCR, TaKaRa LA Taq™ Kit). The primers for GS115–pPIC9K/Latcripin-1 were Latcripin-1—R1seq primer (20 pm) and 5′AOX I primer (20 pm). For GS115-pPIC9K, 3′AOX I primer (20 pm) and 5′AOX I primer (20 pm) were used (Table 2). One characterized clone was selected at random for further work.

### 3.5. Induction and Expression of Protein Latcripin-1 in *Pichia pastoris* GS115

The selected recombinant strains (His+ Mut+) that were integrated with Latcripin-1 and with pPIC9K were cultured in 50 mL YPD medium at 30 °C and 1000 rpm until the OD600 reached approximately 2–6. Then 0.4% of the medium was chosen and transferred into 100 mL BMGY medium, cultured at 30 °C and 1000 rpm until the OD600 reached approximately 2–6. To induce expression of the protein Latcripin-1, 100 mL of the strains were harvested by centrifugation at 4000 rpm for five minutes and then resuspended to approximately 1.5 at OD600 in BMMY. Methanol was added to a final concentration of 1% every 24 h to maintain the induction. One milliliter of supernatants was collected and centrifuged every 24 h. At the time point of 96 h, the supernatants were all collected for the extraction and analysis of the protein Latcripin-1.

### 3.6. Characterization of Protein Latcripin-1 with Western Blot

The supernatants were finally resuspended in 40 μL with 8 μL 1× SDS Sample Buffer, incubated at 95 °C for 10 min, then centrifuged at 10,000 g for 5 min at room temperature. The denatured protein was analyzed by 10% SDS-PAGE. Then, the protein in the gel was transferred to a polyvinylidend difluoride membrane using a semi-dry electroblotting. The membrane was blocked, agitated, with 5% non-fat-milk at 25 °C for 2 h. The first antibody’s (Penta. His Antibody) dilution was 1:1000, and the second antibody’s (HRP-Rabbit Anti-Mouse IgG) dilution was the same. True Blue Peroxides Substrate was selected as the chromogenic substrate. All reaction conditions were at room temperature for 1 h.

### 3.7. Affinity Purification of Protein Latcripin-1 from *Pichia pastoris* Culture Supernatant

The culture supernatants induced for 96 h were freeze-dried and resuspended to 20 mL with deionized water. The Nickel Chelated Column was uncapped and the alcohol was allowed to drain from the gel bed. The gel was balanced by 8 mL binding buffer. Then the 5 mL resuspended protein was added to the column. The flow rate was controlled at 10 mL/h. After that, the column was washed with 15 mL binding buffer. The fractions were collected. Finally, the 6× His-tagged protein was eluted with 5 mL elution buffer and the fractions were collected. The eluted fractions were measured by 10% SDS-PAGE and Microplate reader at 565 nm. The remaining 15 mL resuspended protein was purified with the same method. Then, Bicinchoninic Acid Kit for Protein Determination was used to measure the concentration of the purified protein [43].

### 3.8. Effects of Protein Latcripin-1 on the Proliferation and Apoptosis of Lung Cancer A549 Cells

The Inhibition level of protein Latcripin-1 on A549 cell proliferation was determined by MTT assay. Annexin V-FITC and PI double staining were used to analyze apoptosis. Changes of cell ultrastructure were observed by transmission electron microscopy.

A549 cells were grown in RPMI-1640 medium supplemented with 10% FBS (fetal bovine serum), incubated at 37 °C, in 5% CO_2_ condition in a carbon dioxide incubator.

First, the logarithmic phase of A549 cells was plated in three 96-wells plates at the concentration of 1 × 10^5^ cells/well, and then cultured in 10% FBS RPMI-1640. After 24 h incubation, the experiment was divided into five groups, each of which included three wells. Then the medium was replaced with the same kind of medium, supplemented with different concentrations of protein Latcripin-1 (7.5 μg/mL, 15 μg/mL and 30 μg/mL). Ten percent FBS RPMI-1640 was used as a negative control, while 5.0 μg/mL chemotherapeutics DDP was used as a positive control. After 24 h, 48 h and 72 h of incubation, respectively, the medium was discarded. The cells were incubated with 20 μL 10 mg/mL MTT at 37 °C for 4 h. Then 150 μL DMSO (Dimethyl Sulfoxide) was added per well. The absorbance was detected at 492 nm on a plate reader.

Second, the logarithmic phase of A549 cells was plated in a 25 mL culture flask of 3 × 10^5^ cells/flask, and then cultured in 3 mL 10% FBS RPMI-1640. After 24 h incubation, the medium was replaced with the medium supplemented with 30 μg/mL protein Latcripin-1, 10% FBS RPMI-1640 was used as a negative control. After another 24 h of incubation, half of the cells were trypsinizated with 0.25% EDTA (Ethylenediamine Tetraacetic Acid)-trypsogen and washed with PBS three times. Annexin V-FITC/PI dye was added according to the instruction of the kit. First, 200 μL Binding Buffer was added. Then 10 μL Annexin V-FITC and 5 μL PI were added. After 15 min dark incubation, apoptosis function of protein Latcripin-1 was detected with flow cytometry and analyzed with Cell Quest software [18]. Finally, the other half of the cells were fixed with 2% glutaric dialdehyde and prepared for transmission electron microscopy. After having been fixed at 4 °C for 24 h, the cells were fixed with 1% osmic acid for 3 h. After washing, the cells were dehydrated by being placed in higher and higher concentrations of alcohol at 4 °C. Then, 100% acetone and embedding solution (2:1) were added at 25 °C for 4 h. 100% acetone and embedding solution (1:2) were added at 25 °C for 12 h. Embedding solution was added at 37 °C for 12 h, then incubated at 45 °C for 12 h, and at 60 °C for 24 h. When the embedding medium was hard, the sections were cut on an ultramicrotome. Then the sections were stained with 3% uranyl acetate-lead citrate. The cells’ ultrastructure was observed with electron microscope.

### 3.9. The Toxicity Test of Protein Latcripin-1 on Normal Chick Embryo Fibroblasts

The normal chick embryo fibroblasts were prepared in 96-well plates at a concentration of 1 × 10^5^ cells/well, cultured in 10% FBS RPMI-1640. After 24 h of incubation, the experiment was divided into five groups, each of which included three wells. There were three groups of protein Latcripin-1 (7.5 μg/mL, 15 μg/mL and 30 μg/mL). Ten percent FBS RPMI-1640 was used as a negative control and 5.0 μg/mL DDP was used as a positive control. All of the components were prepared in 10% FBS RPMI-1640. After 24 h, 48 h and 72 h incubation, respectively, the cells were tested with the MTT method at 492 nm.

### 3.10. Statistical Analysis

SPSS10.0 software [44] was used for statistical analysis. Independent-samples T test was used to compare the differences between two groups. *p* < 0.05 was considered to be statistically significant between the two groups.

## 4. Conclusions

In conclusion, a novel apoptosis correlated molecule, protein Latcripin-1 from *Lentinula edodes* C_91–3_, was induced and expressed successfully by using a pPIC9K vector with an alcohol oxidase 1 promoter in *Pichia pastoris* GS115. The protein can induce apoptosis in human lung cancer cells A549. Though further research is required to elucidate the apoptosis mechanism induced by protein Latcripin-1, it is the first time to find and study the new protein (GenBank Accession Number: JQ327159). The work brings new insights and advantages in finding anti-tumor proteins, and addresses the possibilities of solving screening or purification problems, which builds a foundation for further anti-tumor studies of *Lentinula edodes* C_91–3_.

## Figures and Tables

**Table 1:** The modeling parameters and modeling information.

	Modeled Residue Range	Based on Template	Sequence Identity (%)	*E* Value	Modeling Information
FAT	149 to 509	1w3bA	10.11	5.10 × 10^−9^	The superhelical TPR domain of *O*-linked glcnac transferase reveals structural similarities to importin alpha
PI3K	541 to 978	1he8A	17.70	8.10 × 10^−42^	Ras G12V-PI 3-Kinase gamma complex
FATC	1040 to 1070	1w1nA	34.38	4.70 × 10^−9^	The solution structure of the FATC domain of the protein kinase TOR1 from yeast

**Table 2:** Primers used in the experiment.

Primer Name	Primer Sequence (5′→3′)	Length (bp)
3′-RACE Outer Primer 1	TACCGTCGTTCCACTAGTGATTT	23
3′-RACE Outer Primer 2	CACCGGTGTGGAAGGCGTATT	21
3′-RACE Inner Primer 1	CGCGGATCCTCCACTAGTGATTTCACTATAGG	32
3′-RACE Inner Primer 2	TAGATGCCAGGGACCGCTTCT	21
5′-RACE Outer Primer 1	CATGGCTACATGCTGACAGCCTA	23
5′-RACE Outer Primer 2	TCTTCGCCAGGGACCGTAGAT	21
5′-RACE Inner Primer 1	CGCGGATCCACAGCCTACTGATGATCAGTCGATG	34
5′-RACE Inner Primer 2	CTGAGCGTCGGTAGCATTTGC	21
F Primer	CATGGAGAGTGCGAAACTAG	20
R Primer	GAAATGCGGTAAACCACTTG	20
Latcripin-1-R1seq primer	CAATAGGACCACCCAAAACC	20
repair Primer1F	CATGGAGAGTGCGAAACTAG	20
repair Primer1R	GAAATGCGGTAAACCACTTG	20
repair Primer2F	CAAGTGGTTTACCGCATTTC	20
repair Primer2R	ATGTTTGTCCGTCACTCCCG	20
repair Primer3F	CGGGAGTGACGGACAAACAT	20
repair Primer3R	TTGGTGAAGAGGTCCGCGGCTTTG	24
5′-AOX I primer	GACTGGTTCCAATTGACAAGC	21
3′-AOX I primer	GGCAAATGGCATTCTGACAT	20
